# Systematic review of fruit and vegetable voucher interventions for pregnant women and families with young children

**DOI:** 10.1017/S1368980025100657

**Published:** 2025-07-16

**Authors:** Grace Grove, Nida Ziauddeen, Mary Malone, Dianna Smith, Nisreen A. Alwan

**Affiliations:** 1 School of Primary Care, Population Sciences and Medical Education, Faculty of Medicine, University of Southampton, Southampton, UK; 2 NIHR Applied Research Collaboration Wessex, Southampton, UK; 3 King’s College London, London, UK; 4 School of Geography and Environmental Science, University of Southampton, Southampton, UK; 5 University Hospital Southampton NHS Foundation Trust, Southampton, UK

**Keywords:** Fruit and vegetables, Diet quality, Children, Voucher scheme

## Abstract

**Objective:**

This systematic review aimed to explore the impact of food voucher schemes during pregnancy and early life on fruit and vegetable (F&V) consumption and explore experiences of schemes.

**Design:**

Six electronic databases and grey literature sources were searched. Interventional, observational, qualitative and mixed methods studies published from January 2000 to April 2024 in English were included.

**Setting:**

Food voucher interventions targeting F&V intake.

**Participants:**

Low-income pregnant women and families with young children (aged under 5 years).

**Results:**

7344 peer reviewed records and 103 grey literature documents were screened. Sixteen peer reviewed studies (across eighteen reports) and eight grey literature documents met the inclusion criteria. All studies took place in the UK or the USA. There was a lack of consistency across primary quantitative outcomes. Overall, F&V voucher schemes did appear to increase fruit and/or vegetable consumption, but confidence in this finding was low. Qualitative data were more consistent. F&V vouchers were used in three main ways; as a financial benefit to subsidise food already being purchased, to increase the quantity or variety of F&V purchased, or as a safety net, to be used to ensure that the family had something to eat.

**Conclusions:**

F&V vouchers may increase F&V intake and are positively received by recipients. This review also highlights some of the difficulties that researchers face in evaluating the impact of public health measures to improve population health. It is clear that more high-quality research is required to better understand the impacts of F&V vouchers on individual outcomes.

Health, poverty and poor diet quality are inextricably linked. Poor diet quality is linked to many adverse health outcomes, both for children: obesity^(1–3)^, gastrointestinal issues and constipation^(4)^, dental caries^(5,6)^, hypertension^(7)^, diabetes^(7)^ and growth stunting^(8)^ and for pregnant women: gestational diabetes^(9)^, gestational hypertension^(10)^ and excessive weight gain^(11)^. Looking at the impacts from a societal perspective, poor health can result in time away from school or work, increased healthcare costs and losses to the economy. Food insecurity has been associated with increased healthcare costs^(12)^ and poor health outcomes^(13)^. Poverty has been linked with childhood obesity^(14)^, with children from the most deprived decile being twice as likely to be obese as children from the least deprived decile^(15)^.

Maintaining a good quality diet is particularly challenging for those on low incomes. Children from deprived backgrounds are more likely to have poorer diet quality than children from more affluent backgrounds^(16,17)^. In the UK, healthy diets are comparatively more expensive, with F&V costing significantly more per 1000 kcal energy provided (£11·79) than foods and drinks high in fat and sugar (£5·82/1000 kcal)^(18)^. This makes it increasingly difficult for families under financial strain to maintain healthy diets.

F&V vouchers aim to improve dietary quality by safeguarding or increasing spend on F&V in low-income families. They are intended to ensure that families can access F&V that may be out of reach otherwise. Critics may argue that F&V vouchers could be used to offset current spending and could paradoxically decrease diet quality by freeing up money to be spent on unhealthy foods^(19,20)^. Evidence to support interventions such as F&V vouchers can be challenging to gather, and we are not aware of any previous mixed methods reviews that have considered the impact of F&V voucher interventions on the diet and health of pregnant women and families with young children. This review aimed to systematically synthesise published studies (peer reviewed and grey literature) to assess the impact of F&V vouchers on the diets and health of recipients (pregnant women and families with children under the age of 5). The review also aimed to explore recipients’ experiences of F&V vouchers, where F&V voucher schemes face challenges, and what might be done to mitigate these issues.

## Methods

The PICO framework for the review was as follows:

- Population: Low-income pregnant women and families with children under the age of 5, in Organization for Economic Cooperation and Development countries^(21)^, used as a proxy for high-income countries
- Intervention: Means-tested voucher schemes that support healthy diets by at least partly targeting F&V intake.
- Comparator: No voucher scheme, food-based voucher schemes not targeting F&V consumption or non-food-based voucher schemes
- Outcomes:
- Primary outcomes: F&V intake and diet quality.
- Secondary outcomes: F&V purchasing: quantity or expenditure, nutritional value of food shopping, nutritional biomarkers, recipients’ experiences of the scheme and of food shopping, cooking and providing food for themselves or their family, healthcare providers experiences of the scheme, childhood or maternal weight status, breast-feeding rates, maternal diabetes, low or high birthweight, childhood healthcare contacts or healthcare utilisation, parental mental health, expenditure on food and food insecurity.

The protocol for this systematic review was registered on Prospero (PROSPERO 2022 CRD42022364740) on 09/11/2022^(22)^.

### Searches

A search was conducted on six electronic databases: EMBASE (via Ovid), MEDLINE (via Ovid), The Cochrane library, Web of Science, CINAHL (via EBSCO) and IBSS. Searches were restricted to English language articles published from the year 2000 to 30/04/2024. Grey literature searches consisted of grey literature database searches, Google searches and targeted review of specific websites (charitable organisations, think tanks and government bodies). Searches took place on 01/11/2022–03/11/2022 and were updated on 30/04/2024. The full search terms used for Medline was:

[‘healthy start’.mp. or ‘best start’.mp. or WIC.mp. or ‘Farmers Market Nutrition Program’.mp. or ‘women, infants, and children’.mp. or (‘food subsid*’ or ‘food aid’).mp. or voucher*.mp. or coupon*.mp. or (Food Assistance/ or ‘food assistance’.mp.) or ‘fruit* and vegetable* prescription*’.mp. or ‘food buck*’.mp.] AND

[family.mp. or exp Family/ or families.mp. or (‘pre-school’ or preschool).mp. or (exp Infant/ or infant*.mp.) or (Child/ or child*.mp.) or pregnan*.mp. or Pregnant Women/ or parent*.mp. or exp Parents/] AND

[(low adj2 income*).mp. or (exp Poverty/ or poverty.mp.) or exp Socioeconomic Factors/ or depriv*.mp. or disadvantage*.mp. or underprivilege*.mp.]

### Inclusion and exclusion criteria

Interventional, cohort, cross-sectional, case–control, qualitative and mixed methods studies were all included, as well as grey literature with original data from charitable bodies, governmental agencies or think tanks. Conference data, letters and other grey literature were excluded.

One of the most well-known means tested voucher schemes for women and children is the American Special Supplemental Nutrition Program for Women, Infants, and Children (WIC). This US-federal food assistance scheme provides credit to be used to purchase a wide range of food items, designed to improve health by supporting recipients (at-risk and income eligible pregnant women and children under 5 years) to eat a nutritionally balanced diet^(23)^. WIC also includes an educational element. The WIC programme itself does not meet the inclusion criteria for this review, due the large range of foods provided other than F&V and the educational element, However, any ‘add on’ programmes offering additional funds to be spent on F&V to WIC recipients, with no compulsory educational elements do meet inclusion criteria.

### Screening process and data extraction

A sample of titles (10 %) was reviewed independently for inclusion by GG and NZ. Good agreement was achieved (> 80 %) and GG then independently screened the remainder of the titles. A sample of abstracts for the selected titles (10 %) was then reviewed independently by GG and NZ, following the same process as that employed for the titles as good agreement was again achieved. Finally, GG and NZ independently reviewed all full-text articles selected by abstract screening.

Two authors (GG and NZ) independently undertook data extraction on a 20 % sample and discussed any discrepancies. No significant discrepancies were found, and GG then completed data extraction on the remaining papers. Data were extracted using a standardised form and included funding information, study location, study design, inclusion/exclusion criteria, recruitment method, population studies, sample size, demographic information of participants (age, sex, ethnicity and socio-demographic information), intervention (including type, duration and cost), outcomes (including timepoints measured), analysis methods, loss to follow-up and main findings. Any relevant outcome data were collected and recorded as presented in the original paper.

### Risk of bias assessment

Risk of bias assessment was undertaken by GG and NZ, who reviewed papers independently and then discussed quality to come to an agreed final assessment. Appraisal tools were selected depending on study type, non-randomised interventional and cohort studies were assessed using Cochrane ROBINS-I tool^(24)^, cross-sectional studies using the National Heart, Lung and Blood Institute Quality Assessment for Observational Cohort and Cross-sectional Studies tool^(25)^ and case control and qualitative studies using Critical Appraisal Skills Programmes checklists^(26)^. Grading of Recommendation, Assessment, Development and Evaluation^(27)^ was used to assess certainty of evidence^(28,29)^ across all quantitative outcomes included in this review, where more than one paper contributed findings.

### Data synthesis

As expected, there was considerable heterogeneity in the papers selected for inclusion in the review as mixed methods, quantitative and qualitative studies all met inclusion criteria. As such, it was not possible to undertake meta-analysis or other synthesis methods and a formal narrative approach to data synthesis was utilised, following Popay et al.’s Guidance on the Conduct of Narrative Synthesis in Systematic Reviews (2006)^(30)^. Following the research protocol, studies were grouped by study type (quantitative, qualitative and mixed methods) and then by intervention type (for example, Healthy Start (HS) in the UK).

PRISMA guidelines were followed to ensure transparent reporting of data synthesis^(31)^. Vote counting and concept mapping were used to synthesise the data and to explore relationships between data.

### Outcome measurement

The primary outcome of interest in this review is F&V intake and diet quality. The outcomes and definitions used in the original paper have been used in this review. Measures of diet quality may include portions of F&V consumed, deduced from diet recall, food diaries and FFQ, as well as dietary and nutrient intakes calculated from food diaries and FFQ, or other assessments of diet quality.

## Results

**Figure 1.**
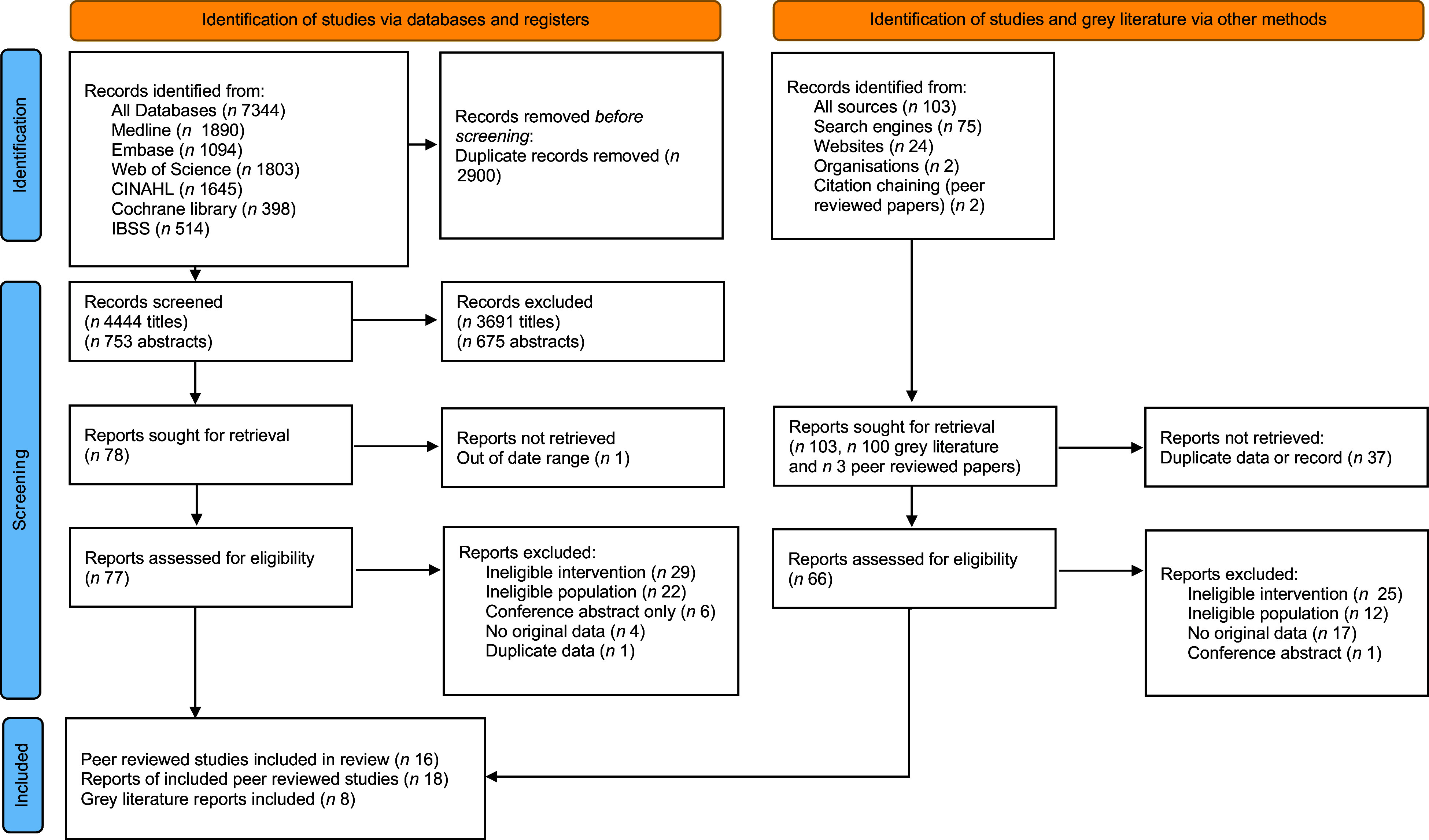
PRISMA 2020 flow diagram.

Screening was documented using a PRISMA flow diagram (Figure 1)^(31)^. Searches identified 7344 records from databases and registers, including 2900 duplicate records. 4444 titles and 753 abstracts were screened for inclusion. Full-text reports (*n* 77) were then assessed for eligibility, with sixteen studies (*n* 18 reports) included^(20,32–48)^. Of these, nine studies (*n* 10 reports)^(20,39–47)^ were in the UK and seven studies (*n* 8 papers)^(32–38,48)^ in the USA. Four studies were non-randomised trials^(32–35)^, one was a cohort^(36)^, three were cross-sectional^(20,37,38)^, four were before and after^(39–41,47)^, five were qualitative^(42–45,48)^ and one was mixed methods^(46)^.

In total 103 grey literature records were identified, thirty-seven of which were duplicates, and thus sixty-six records were assessed for eligibility. Eight grey literature reports were included in this review^(49–55)^.

### Schemes included

Within this review, a range of programmes have been explored, including HS, Rose vouchers, Sainsbury’s top up vouchers and Best Start foods (BSF) schemes in the UK and add-on programmes linked to WIC (including the Farmers’ Market Nutrition Programme (FMNP) and EatSF) in the USA. Detailed information on each of the schemes is provided in Table 1. HS replaced the previous Welfare foods Scheme in 2006^(39)^, first with a paper-based scheme and more recently a digital scheme^(56)^. The paper-based scheme provided vouchers that could be used to purchase fruits, vegetables, cow’s milk or formula^(57)^. The digital HS scheme^(56)^ has increased value (£4·25 per week) and includes a wider variety of foods that can be purchased. In Scotland, HS has been replaced with BSF^(58)^. Welfare foods Scheme and HS/BSF are government-funded schemes. BSF is similar to HS, with some differences in eligibility criteria, foods permitted and a higher voucher value. Sainsbury’s is a UK supermarket chain that provided additional vouchers to HS redeemers as part of its food donation programme, which could be redeemed against fresh or frozen F&V only for 6·5 months in 2021^(59)^. Finally, the Rose voucher scheme^(60)^ is run by a charity and provides vouchers that can be exchanged for fresh fruit and vegetables but only operates in some parts of the UK. In the USA, the FMNP provides recipients of the US-federal WIC food assistance program with additional vouchers that can be used to purchase F&V from farmers’ markets and roadside stalls. Similar to WIC, exact benefits vary from state to state, and FMNP currently operates in forty-nine states^(61)^. The food costs and 70 % of administrative costs of FMNP are supported through federal funding to state agencies. EatSF was funded through the Department of Public Health, City and County of San Fracisco and other supporters and provides pregnant WIC recipients in San Francisco with additional vouchers to spend on F&V^(62)^.

**Table 1.** Fruit and vegetable voucher schemes included in this review

Scheme name	Location	Scheme details
Welfare Food Scheme (WFS)	UK	Historic scheme (1940–2006), replaced by Healthy Start Provided milk, infant formula and vitamins to pregnant women, new mothers and young children
Paper-based Healthy Start (HS)	UK	Historic scheme (2006–2021), replaced by digital HS Provided paper vouchers which could be spent on fruits, vegetables, cow’s milk or formula as well as pulses and beans from 01/10/2020 onwards. Eligible persons received £3·10 (pregnant women and children aged 1–4 years) or £6·20 (infants aged 0–1 year) per week^(57)^. Scheme provided free vitamins, separately to the vouchers^(56)^.
Digital HS^(56)^	England, UK	Prepaid card for pregnant women (over 10 weeks gestation) and children aged up to 4 years, which can be spent on fruit, vegetables, cow’s milk, infant formula, pulses and beans. Income assessed and eligible persons receive £4·25 (pregnant women and children aged 1–4 years) or £8·50 (infants aged 0–1 year) per week. Scheme provides free vitamins, separately to the vouchers.
Rose Vouchers^(60)^	UK	Available in some parts of the UK. Vouchers for fresh fruit and vegetables (F&V) for low-income families with children aged 4 and under – £6/week for children aged 0–1 year and £4/week for children aged 1–4 years.
Sainsbury’s top up vouchers^(59)^	England, UK	Historic scheme (15th February 2021–30th August 2021) Checkout tills automatically printed a voucher for additional £2 to spend in stores on fresh and frozen F&V when shoppers redeemed a HS voucher.
Best Start Foods (BSF)^(58)^	Scotland, UK	Prepaid card for eligible pregnant women and children under 3, which can be used to in store or online to purchase F&V, milk, formula, pulses and eggs. Pregnant women and children aged 1–3 years receive £4·95/week, and infants from 0–1 receive £9·90/week.
Farmers’ Market Nutrition Programme (FMNP)^(61)^	USA	Special Supplemental Nutrition Program for Women, Infants, and Children (WIC) recipients provided with additional vouchers that can be used to purchase F&V from farmers’ markets and roadside stalls, exact benefits vary from state to state.
EatSF^(62)^	USA	Additional vouchers for pregnant WIC recipients to spend on F&V, the amount received varies.

### Peer reviewed papers

Table 2 summarises the characteristics of the included papers including strengths and limitations.

**Table 2.** Summary of included peer reviewed papers

Paper ID	Study design, location and recruitment period	Study population and sample size	Study population summary statistics										Intervention	Relevant outcomes included	Strengths(+) and limitations(-)
Primarily quantitative studies															
Herman 2006^*(32)^	Non randomised trial^§^ Repeated interviews. USA February–August 2001	Post-partum women on the Special Supplemental Nutrition Program for Women, Infants, and Children (WIC) *n* 602	Age (years), mean (range)						27·2 (17–43)				Intervention: $10/week in vouchers to spend on fruit and vegetables (F&V), at a supermarket (SM) or farmer’s market (FM). Control: $13/month in vouchers to spend on diapers.	F&V purchasing	+ Interviews undertaken by trained professionals + Followed up over time + Collected data on reasons for drop outs and unspent vouchers - Dropout rate 25 % (148/602) - No individual level analysis - No adjustment for confounding - Did not present any data relating to control group - Differences in some data compared with other paper published using the same cohort^(33)^
			Ethnic background, %	Hispanic					86·3						
				Non-Hispanic black					6·6						
				Non-Hispanic white					3·9						
				Asian American					3						
				Native American					0·2						
Herman 2008^*(33)^	Non randomised trial^§^ Repeated interviews. USA February–August 2001	Post-partum women receiving WIC *n* 602	Age (years), mean (range)						27·5 (17–43)				As for Herman et al 2006.	F&V consumption	+Adjusted for multiple comparisons + Assessed for continued impact after the end of the intervention + Adjusted for confounders - Dropout rate 25 % (151/602) - Differences in some data compared with other paper published using the same cohort^(32)^
			Ethnic background, %	Hispanic					89·1						
				African American					5·9						
				Non-Hispanic white					2·8						
				Asian American					1·9						
				Native American					0·2						
Ridberg 2020^†(34)^	Non randomised trial§ Self-administered surveys. Taking advantage of introduction of supplementary voucher for pregnant women receiving WIC. USA February–August 2017	Intervention: pregnant and (up to 5 months) post-partum women claiming WIC: *n* 592 Control: non pregnant WIC participants: *n* 108 Preterm birth outcome only: historical control from birth records in the previous year’s WIC cohort	Demographics for intervention group only, *n* 592										Intervention: EatSF. Control: normal WIC benefits and $10 drug store voucher.	F&V consumption Food security (6 item USDA food security survey module) Preterm birth (using historical control group)	+ Included English, Spanish and Chinese speakers + Used validated food security measure + Takes advantage of natural experiment - Differences between intervention and control groups - No demographic data for control group - High loss to follow-up (32 % in intervention group) - Time to follow-up different in intervention and control groups
			Age (years), mean (range)						30 (16–43)						
			Ethnic background, %	Asian					278 (55 %)						
				Hispanic/ Latino					168 (33 %)						
				White, not Hispanic					18 (4 %)						
				Black or African American, not Hispanic					35 (7 %)						
				Multi racial					6 (1 %)						
				Native Hawaiian or Other Pacific Islander					5 (1 %)						
Ridberg 2022^†(35)^	Non randomised trial§. Self-administered surveys. USA September 2020–June 2021	Intervention: pregnant women claiming WIC in San Francisco: *n* 304 Control: pregnant women claiming WIC in neighbouring counties: Alameda *n* 226 and San Mateo *n* 240			Intervention *n* 304				Control *n* 466				Intervention: EatSF Control: normal WIC benefits. All participants received a $20–$30 retail voucher per completed survey	F&V consumption Food security (6 item USDA food security survey module)	+ Included English, Spanish and Chinese speakers + Used validated food security measure + Undertook some sensitivity analyses - Some overlap with national policy change that introduced more vouchers for F&V for all WIC claimants - Some loss to follow-up (21 %) - Undertaken during COVID pandemic which may have impacted on shopping habits and ran concurrently with other pandemic related interventions - Differences between groups at baseline not controlled for
			Age, years, *n* (%)	18–25	68 (22 %)				143 (31 %)						
				26–35	177 (58 %)				257 (55 %)						
				36–45	58 (19 %)				60 (13 %)						
				> 45	1 (0 %)				0 (0 %)						
				Prefer not to answer	0 (0 %)				6 (1 %)						
			Ethnic background, *n* (%)	Black/African American	31 (11 %)				55 (12 %)						
				Asian/Pacific Islander	106 (36 %)				58 (13 %)						
				White/Caucasian	15 (5 %)				31 (7 %)						
				Native American/American Indian	0 (0 %)				4 (<1 %)						
				Other	2 (1 %)				6 (1 %)						
				Prefer not to answer	4 (1 %)				16 (4 %)						
Wang 2022^†(36)^	Cohort§. Secondary data analysis of birth records. USA 2009–2019	Total sample: *n* 1, 831, 649 records. Sample in intervention county *n* 19, 861 (approximately 2, 200 of these enrolled in programme) Sample in control counties *n* 1, 811, 788			Intervention *n* 19, 861 (∼2, 200 enrolled in programme)				Control *n* 1, 811, 788				EatSF.	Low birth weight (< 2500 g) Preterm (< 37 weeks) Small for Gestational Age (SGA) Maternal Gestational Diabetes Mellitus (GDM) Weight gain (within, above or below guidelines)	+ Adjusted for confounders where possible + Used synthetic control group + Large study making use of available data - Only a small proportion (∼11 %) of the intervention sample were likely to have actually received the intervention, this could not be verified. - Data error in dataset meant that some participants were mis-classified, which lead to large number of records being removed from the dataset - Some missing data
					Pre *n* 15 503		Post *n* 4358		Pre *n* 1 374 512		Post *n* 437 276				
			Age (years), mean		28·6		29·4		26·7		27·7				
			Ethnic background, n	Non-Hispanic White	7·6		5·8		12·6		11·6				
				Non-Hispanic Black	10·4		9·0		7·1		6·6				
				Hispanic	49·3		51·9		73·4		74·0				
				Other non-Hispanics	32·7		33·4		7·0		7·9				
Blumberg 2022^(37)^	Cross-sectional||. In person survey data collected from WIC offices. USA October 2017–January 2018	WIC claimants who also received Farmers’ Market Nutrition Program (FMNP) vouchers. *n* 329	Age, *n* (%)	< 24					62 (18·9)				FMNP $20 in coupons for F&V/claimant/ season.	F&V consumption Barriers and facilitators to FM use (open question survey data) Food purchasing habits (open question survey data)	+Population of confirmed FMNP recipients + Included non-redeemers - Self-reported and recall data - Convenience sampling - No adjustment for confounders
				25–34					155 (47·1)						
				> 35					100 (30·4)						
				Missing					12 (3·7)						
			Ethnic background, *n* (%)	Asian/pacific islander					5 (1·5)						
				Black or African American					50 (15·2)						
				Hispanic or Latino					239 (72·6)						
				Native American					1 (0·3)						
				White					13 (4·0)						
				Other					6 (1·8)						
				Missing					15 (4·6)						
Kropf 2007^(38)^	Cross-sectional||. Postal survey for WIC claimants, some of whom were receiving FMNP in addition to WIC. USA November 2005	Female household contact for WIC receiving either WIC alone, or WIC plus FMNP. WIC alone: *n* 170, WIC plus FMNP: *n* 65	Age and ethnic background not reported										FMNP $18 in coupons for F&V/claimant/ season.	F&V consumption Food security FMNP participation, satisfaction and behaviour (closed question survey data)	+Population of confirmed WIC/FMNP recipients + Had comparator group - Low response rate (WIC = 20·4 %, WIC + FMNP = 26·4 %) - No adjustment for confounding - Limited demographic data for participants - Participants self- selected
Parnham 2021^(20)^	Cross-sectional||. Secondary data analysis of Households (HH) that were eligible or non-eligible for Healthy Start (HS), using annual Living Costs and Food Survey. England, UK 2012–2017	HH with a pregnant women or child aged 0–3, in four groups, eligible (EP), eligible non (ENP), nearly eligible (NE) and ineligible (I) HH *n* 4869	All demographics for the HH representative										Paper-based HS	F&V expenditure and quantity HS foods expenditure and quantity Infant formula expenditure Total food expenditure	+Detailed expenditure data (2 weeks) + Income and expenditure data confirmed with supporting documents + Appropriate analysis and adjusted for confounders - - Small number of participants eligible for HS - Response rate < 50 %
					EP (*n* 475)		ENP (*n* 401)		NE (*n* 428)		I (*n* 3565)				
			Age, years		31·1		32·8		33·3		35·8				
			Ethnic background, *n* (%)	White	400 (84)		340 (84·8)		313 (73·1)		3047 (85·5)				
				Ethnic minority	76 (16)		61 (15·2)		115 (26·8)		518 (14·5)				
Ford 2008^‡(39)^	Before and after§. Natural experiment investigating impact of policy change, removal of welfare food scheme (WFS) and introduction of HS. Data collection by trained interviewers England, UK WFS: November 2005–2006 HS: April-November 2007	Caucasian pregnant (P) and postpartum (PP) women who may have been eligible for WFS or HS (determined using proxy measures). WFS: Pregnant: *n* 90, PP: *n* 86 HS: Pregnant: *n* 96, PP: *n* 64 Total *n* 336			WFS P (*n* 90)		HS P (*n* 96)		WFS PP (*n* 86)		HS PP (*n* 86)		WFS and paper-based HS scheme	Food consumption including F&V Nutrient intake	+ Takes advantage of natural experiment + Data collected by trained interviewer + Adjustment for some confounding - Large differences in under/over reporting between groups - Used FFQ rather than weighted food diary - Excludes non-Caucasian, non-English speaking women
			Age, years		22		21·5		25		22				
			Ethnic background, (%)	Caucasian	100		100		100		100				
Griffith 2018^(40)^	Before and after§. Secondary data analysis of UK shopping data (Kantar) before and after HS introduction. England, UK December 2004–November 2008	Low-income HHs, split into likely eligible for HS (determined using proxy measures) and non-eligible (children aged 4–8 years, women prior to a pregnancy) *n* 296 HH	Demographic data not available										Paper-based HS	Spend on F&V Quantity of F&V purchased Nutritional composition of shopping baskets	+ Takes advantage of natural experiment + Robust analysis of extensive dataset + Controlled for some confounding and tested for robustness as possible with the available data - Inadequate data on HH income/benefits available in the dataset so used hours worked as proxy - Unclear how closely proxies correlate with variable of interest. Likely that underlying assumptions mis-identify some participants - Food purchases rather than consumption
Mouratidou 2010^‡(41)^	Before and after§. Natural experiment investigating impact of policy change, removal of WFS and introduction of HS. Data collected by trained interviewer across three time points (baseline, 8 and 12 weeks PP). England, UK WFS: November 2005–November 2006 HS: April–November 2007	Caucasian postpartum women who may have been eligible for WFS or HS (determined using proxy measures). WFS: *n* 86 women HS: *n* 64 women Total *n* 150			WFS *n* 86				HS *n* 64				WFS and paper-based HS	Food consumption including F&V Nutrient intake	+ Takes advantage of natural experiment + Data collected by trained interviewers + Adjustment for some confounding - Large differences in under/over reporting between groups - Used FFQ rather than weighted food diary - Excludes non-Caucasian, non-English speaking women −58·7 % of WFS and 64·5 % of HS group did not provide data at all three time points - Presents unadjusted values
			Age, years		25				22						
			Ethnic background, (%)	Caucasian	100				100						
Thomas 2023^(47)^	Before and after§. Single-arm intervention trial investigating impact of supermarket top up vouchers for HS recipients, using supermarket loyalty card data. England, UK February–August 2021	Longitudinal analysis: HH receiving and redeeming at least one top up voucher in the study period, and also made purchases in 2019 and 2020. *n* 133 Cross-sectional analysis: HH receiving top up vouchers in intervention period (redeemers and non-redeemers) *n* 150			Longitudinal analysis *n* 133				Cross- sectional analysis *n* 150				Top up supermarket vouchers for fresh and frozen F&V for HS recipients	Total food spend F&V spend F&V weight F&V portions Proportion of F&V in basket Proportion of different types of F&V in basket	+ Takes advantage of natural experiment + Detailed basket information available + Both longitudinal and cross-sectional analysis undertaken + Redeemers and non-redeemers included - Presents unadjusted values (unable to adjust due to limited data available) - More than half the longitudinal analysis sample only had one ‘redeeming’ shopping basket in the study period. - only a small number of the total recipients of the top up vouchers were included
			Age group, years, *n* (%)	18–34	37 (27·8)				41 (27·3)						
				35–44	34 (25·6)				39 (26·0)						
				45–54	19 (14·3)				23 (15·3)						
				55–64	21 (15·8)				23 (15·3)						
				65+	22 (16·5)				23 (15·3)						
				Unknown	0 (0)				1 (0·7)						
Qualitative papers															
Lucas 2015^(42)^	Qualitative¶. In depth interviews with purposively recruited parents. England, UK 2011–2012	Parents and guardians: *n* 107	No demographic details available										Paper-based HS	Participants experiences of HS, including those who were eligible but not receiving the vouchers.	+ Purposive sampling and a large sample size resulted in a range of views from different participants + Included those who were eligible but not receiving HS, and applicants not in receipt - Some information is lacking- particularly around recruitment and study processes - Couldn’t include families that were not in contact with healthcare services
McFadden 2014^(43)^	Multi-method qualitative¶. Focus groups and online consultation with healthcare professional (HCP), workshops, focus groups and interviews with low-income parents. England, UK March 2011–April 2012	HCP participating in workshops: *n* 49 Online consultation: *n* 619 Parents: *n* 113	Demographic details for parent participants *n* 113										Paper-based HS	Participants and HCP experiences of HS.	+ Large sample + Views from HCP and parents + Different methods for different groups of participants - Limited numbers of young mothers in sample - Missing data for some participants - Some lack of clarity in the reporting of the methodology and data
			Age (years), *n* (%)	≤ 20					12 (11·1)						
				21–30					56 (51·3)						
				31–40					34 (31·2)						
				> 40					4 (3·7)						
				Missing					3 (2·8)						
			Ethnic background, *n* (%)	White British					43 (39·4)						
				White other					8 (7·3)						
				Asian					30 (27·5)						
				Black					20 (18·3)						
				Arab					1 (0·9)						
				Mixed					2 (1·8)						
				Other					5 (4·6)						
Moonan 2022^(44)^	Qualitative¶. Interviews with parents and HCP England, UK February–September 2012	Parents *n* 25, HCP *n* 11, Commissioners and HS staff *n* 6	No demographic details available										Paper-based HS	Participants and HCP experiences of HS.	+ Pilot work informed the topic guide + Triangulated data from different groups - HCP, commissioners and HS staff and parents - No demographic data included - Parents self-identified as being eligible for HS
Ohly 2019^(45)^	Qualitative¶. Realist interviews with parents England, UK September 2016–May 2017	Pregnant and PP women: *n* 11	Age (years), *n*	18–25					7				Paper-based HS	Participant’s experiences of HS.	+ Considers context and motivators behind behaviours + Detailed data available for study participants + Different approach to other similar work, allowing new insight - Realist methods may influence participants responses (researcher shares their views) - Small sample size and no ethnic diversity - Recruitment rate 5 % - Data saturation not reached - Unclear why some participants were invited to second interviews and others were not
				26–35					4						
			Ethnic background, *n*	White British					11						
Jacobs 2023	Qualitative¶. Semi-structured interviews. USA July–December 2021	WIC claimants who also received FMNP *n* 11 WIC and Farmer’s market staff *n* 10	No demographic details available										FMNP	Participant and staff experiences of FMNP.	+ Four researchers coded data and discussed to reach consensus on findings + Included non-redeemers - Limited data on participants included - Limited detail included on methods used
Mixed methods studies															
Dundas 2023^(46)^	Mixed methods§,¶. Secondary data analysis of linked data sets. Semi-structured interviews UK Quantitative data: 2010–2011 Qualitative data: 2015-unclear	Quantitative: Participants from two surveys (Growing up in Scotland (*n* 6127) and Infant feeding survey (*n* 10 768) Intervention: women receiving HS Control: Eligible, not receiving HS Nearly eligible Qualitative: Mothers from low-income backgrounds, some receiving HS *n* 40	Age, years, *n*	20–29					15				Paper based and digital HS	Breast-feeding initiation and duration Experiences of the scheme Low birth weight Maternal mental health	+ Makes pragmatic use of existing datasets + Robust analysis of data accounting for confounding where possible + Some purposive sampling for qualitative data resulting in participants from a range of backgrounds - Had to use difference analysis methods for the different datasets - some participants has used HS vouchers < 5 years previous to the interview date
				30–39					16						
				≥ 40					9						
			Ethnic background, *n*	White					33						
				Black African					3						
				Asian					4						

Scheme details: please see Table 1 for details of the schemes included.

^*, ‡^Indicates associated papers, same study population.

^†^Indicates associated papers, different study populations.

§ Assessed using the ROBINS -I tool, which results in a rating of low risk of bias, moderate risk of bias, serious risk of bias, critical risk of bias or no information.

|| Assessed using the Quality Assessment Tool for Observational Cohort and Cross-Sectional Studies, which results in a rating of good, fair or poor.

¶ Assessed using the CASP checklist for qualitative studies, which does not give specific scoring.

### Quantitative papers

#### F&V intake, diet quality, food purchasing, portions of fruit and vegetables and nutrient intake

Eleven papers considered the impact of F&V vouchers on either food consumption (most commonly F&V intake)^(33–35,37–39,41)^, nutrient intake^(39,41)^ and/ or food purchasing^(20,32,40,47)^ (Table 3). Seven studies examined the impact of F&V vouchers on food consumption and nutrient intake. Four studies found F&V vouchers were associated with an increased intake of F&V combined^(33,34,39,41)^, and two with increased intake of vegetables alone^(37,38)^. One study found no differences in F&V intake between the intervention group receiving $40 voucher for F&V and the control group^(35)^.

**Table 3.** Studies considering the impact of fruit and vegetable vouchers on fruit and vegetable intake, diet quality, nutrient intake and food purchasing

Paper ID and scheme	Main findings	Fruit and vegetable (F&V) purchasing	F&V consumption	Vegetable consumption	Factors that may have influenced results
Herman 2006^*(32)^, FMNP	10 most frequently bought items: oranges, apples, bananas, peaches, grapes, tomatoes, carrots, lettuce, broccoli and potatoes	N/A	–	–	N/A - descriptive results only
Herman 2008^*(33)^, FMNP	Participants from both FM and SM sites reported consuming more F&V (FM: 7·8 and SM: 8·2 servings/2000 kcal) than control (6 servings/2000 kcal, *P* =< 0·001) Higher consumption maintained after 6 months compared with control (7·5 for FM, 7·4 for SM and 4·9 servings/2000 kcal for control, *P* = 0·001) Increase in servings primarily driven by vegetable consumption F&V intake increased by 2·8 servings/2000 kcal (*P* < 0·001) in participants at FM site at end of intervention, then decreased to 2·3 servings/2000 kcal at 6 months post intervention (*P* < 0·001) Increased F&V consumption from baseline to follow-up in the SM site not statistically significant		↑	↑	Differences between groups but some adjustment for confounding and for multiple estimates
Ridberg 2020^†(34)^, EatSF	Intervention group showed greater increases in F&V consumption frequency: Total vegetable (0·59 times/day, *P* < 0·01) Combined F&V (0·73 times/day, *P* < 0·05) Salad (0·23 times/day, *P* < 0·01) Non-fried potato (0·19 times/day, *P* < 0·01) Fruit juice (0·27 times/day, *P* < 0·01)		↑	–	Unadjusted estimates (difference in difference) Differences between intervention and control groups with no adjustment for confounding Time to follow-up different
Ridberg 2022^†(35)^, EatSF	Intervention group ate more F&V at baseline No significant differences in F&V consumption between groups at follow-up		–	–	Unadjusted estimates (difference in difference) COVID pandemic led to altered shopping habits and additional assistance schemes running concurrently with the intervention. This may have resulted in dilution of impact of the intervention and altered shopping habits.
Blumberg 2022^(37)^, FMNP	‘Redeemers’ ate more servings of vegetables (1·66 *v*. 1·43 portions, *P* = 0·050)		–	↑	Unadjusted estimates, no adjustment for confounders
Kropf 2007^(38)^, FMNP	WIC plus FMNP had significantly more vegetable servings per day than WIC alone (2·23 *v*. 1·91, *P* = 0·04, unadjusted) No significant differences in fruit consumption FMNP had higher scores for perceived benefit and perceived diet quality		–	↑	Unadjusted estimates, no adjustment for confounders Self-selecting participants
Parnham 2021^(20)^, HS	475/876 (54·2 %) eligible households claimed HS No statistically significant differences between HS participants and eligible non-participants in HS food or total food expenditure or quantity HS participants spent significantly less on infant formula (− £1·82 /week; 95 % CI –3·12, −0·51) than eligible non-participants. Nearly eligible and ineligible HH spent more on HS foods than eligible non-participants (£1·60, 95 % CI 0·79, 2·41 and £2·56 95 % CI 1·77, 3·35), respectively.	–			Small number of participants that were eligible for HS Adjusted estimates
Ford 2008^‡(39)^, HS	Both pregnant and postpartum (PP) women in HS group had increased nutrient intakes (energy, Ca, folate, Fe and vitamin C) compared with the WFS group Pregnant woman in the HS group consumed more F&V/day (3·3 portions) than pregnant women in the WFS group (2·5 portions, *P* = 0·004) PP in the HS group consumed more F&V/day (3·3 portions) than PP in the WFS group (2·7 portions, *P* = 0·023) Some differences attributed to increased food intake in HS group		↑	–	Differences in over/ underreporting between groups Some adjustment for confounding
Griffith 2018^(40)^, HS	£2·43 increase in spending on F&V/month (15 % increase) £1 of HS vouchers results in £0·14 increase in spending on F&V Increase in F&V quantity of 1·79 kg per month Levels of fibre, vitamin A, Fe and carbohydrate in food shopping increased, fat and sugar did not No indication that purchases of ‘unhealthy’ foods were increased	↑			Used proxies to classify intervention status Unadjusted estimates (difference in difference)
Mouratidou 2010^‡(41)^, HS	HS women had higher intakes of all key nutrients (energy, protein, fat, carbohydrate, fibre, Ca, Fe, Zn, folate and vitamin C) at all time points HS consumed more F&V than WFS: first follow-up 4·1 *v*. 2·8 portions, second follow-up 3·7 *v*. 2·7 portions		↑	–	Differences in over/ underreporting between groups Unadjusted estimates
Thomas 2023^(47)^, Sainsbury’s top up vouchers	F&V purchases increased by weight and by spend across the three time periods. Proportion of spend on F&V increased between 2020 and 2021, but not between 2019 and 2020. HH bought more portions of F&V in 2021 compared with 2020 and 2019 (0·9 portions/day in 2021 compared with 2019, *P* = 0·007) Redeeming baskets contained more portions of F&V. This appeared to be driven by increased purchases of fruit Total spend was increased in the intervention group	↑			Small dataset compared with number of recipients More than half the sample only had one ‘redeeming’ shopping basket in the study period Unadjusted estimates

*, ‡ indicates associated papers, same study population. † indicates associated papers, different study populations.

#### Healthy Start

Two studies found that HS was associated with increased F&V intake and increased macro and micro nutrient intakes^(39,41)^. When considering the impact of F&V vouchers on food purchasing, Griffith et al. used purchasing data from a panel of households in the UK to examine differences between households that were likely and not likely to be eligible for HS vouchers^(40)^. They found that £1 of HS vouchers resulted in £0·14 increase in spending on F&V^(40)^. Levels of fibre, vitamin A, Fe and carbohydrate in food purchases made in eligible households increased, whilst fat and sugar did not^(40)^. In contrast, Parnham et al. found no significant differences in F&V purchasing when comparing HS participants with eligible non-participants^(20)^. Thomas et al. explored Sainsbury’s top up vouchers distributed to HS recipients using loyalty card data^(47)^. They found that recipients spent more on F&V and bought more portions of F&V in the intervention period compared with the control period. They also found that those who redeemed the vouchers purchased more F&V than those who did not^(47)^.

#### Farmers Market Nutrition Program and EatSF

Herman et al. compared supermarket and farmers’ market vouchers for F&V and found that there was increased F&V servings at both intervention sites (primarily driven by vegetable consumption)^(33)^ compared with the control group. The differences in F&V consumption at the intervention sites (both the farmers market’ and supermarket sites) compared with the control group (who received diaper vouchers) remained statistically significant 6 months after the end of the intervention^(33)^. F&V consumption by individual participants over time was also significantly increased in both the farmers’ market and supermarket groups, but this only remained significant in the farmers’ market group at follow-up^(33)^. Two further studies examining the FMNP found the programme to be associated with increased vegetable intake, but not with increased fruit intake^(37,38)^. Two studies by the same group exploring EatSF found contrasting results, with the pilot study (recruitment from February to August 2017, 700 participants) reporting increased F&V consumption frequency^(34)^, but a later study (recruitment from September 2020 to June 2021, 770 participants) finding no significant differences^(35)^.

In the USA, Herman et al. also looked at food purchasing and found that the items most frequently bought with F&V vouchers were oranges, apples, bananas, peaches, grapes, tomatoes, carrots, lettuce, broccoli and potatoes^(32)^.

#### Food security

Three papers considered food security as an outcome^(34,35,38)^ (Table 4), all in the USA exploring EatSF^(34,35)^ and FMNP^(38)^. Ridberg et al. explored EatSF in San Francisco, where pregnant women were automatically enrolled in EatSF whilst attending a pregnancy WIC appointment. They formed a control group of non-pregnant women who were receiving standard WIC benefits. Ridberg et al. report that significantly more women in the intervention group were food insecure at baseline (53 % *v*. 38 %), and amongst those who were food insecure, more women in the intervention group became food secure at 3 month follow-up than in the control group (23 % *v*. 14 %, *P* = 0·04, unadjusted estimate)^(34)^. In their later work, Ridberg et al. found no significant difference in food security status between pregnant women receiving WIC and EatSF, and pregnant women in neighbouring counties receiving WIC alone^(35)^. Kroft et al. sent postal surveys to female head of household registered for WIC in Athens county, Ohio, USA, where the FMNP was available to all WIC recipients^(38)^. They found no significant differences in food security (using unadjusted estimates) between those receiving WIC alone and those receiving WIC and FMNP^(38)^.

**Table 4.** Studies considering the impact of fruit and vegetable vouchers on food security, breast-feeding, low birthweight and maternal outcomes

Paper ID	Food security	Breast-feeding	Low birthweight	Maternal outcomes
Kropf 2007^(38)^	No significant differences in food security between groups, unadjusted estimates	–	–	–
Ridberg 2020^*(34)^	Significantly more women in the intervention group were food insecure at baseline (53 % intervention group *v*. 38 % control group) Amongst women who were food insecure at baseline, more women in the intervention group became food secure at follow-up compared with women in the control group (23 % *v*. 14 %, *P* = 0·04) Using a continuous measure of food insecurity, food insecurity in the intervention group decreased from 3·32 at baseline to 2·32 at follow-up. In the control group, food insecurity decreased from 2·5 at baseline to 2·4 at follow-up. Mean difference in change in score was 0·88 (*P* < 0·001) Unadjusted estimates	–	–	–
Ridberg 2022^*(35)^	No significant differences in food security between groups at follow-up, some adjustment for time varying confounding	–	–	–
Dundas 2023^(46)^	–	Infant Feeding Study (IFS): R were less likely to have ever breastfed than E (57 % *v*. 69 %, *P* < 0·0001) Duration of breast-feeding was less in R than E (average 1·37 months *v*. 1·94 months, *P* < 0·0001) Growing Up in Scotland (GUS): No significant differences in breast-feeding rates or duration of breast-feeding Regression discontinuity analysis for GUS: There were no significant differences in breast-feeding between groups R and E. Those in group R breastfed for 0·45 months less than those in NE.	IFS: No significant difference in low birth weight (< 2500 g) between R and E, significantly fewer low birth weight infants in NE compared with R (0·052 % *v*. 0·071 %, *P* = 0·025) GUS: No significant difference in the rate of low birthweight Regression discontinuity analysis for GUS: There were no significant differences in birthweight between groups R and E. Those in group R were more likely to have low birth weight infant than those in NE.	GUS: No significant differences in maternal mental health between R and E. NE had significantly better mental health scores using SF-12 questionnaire (R = 50·69 *v*. NE = 52·28, *P* = 0·0045)
Wang 2022^(36)^	–	–	No significant differences in low birth weight	No significant differences found in any outcome examined (maternal gestational diabetes and maternal weight gain).

* indicates associated papers, same study population.

#### Health outcomes

Two papers explored differences in health outcomes (Table 4)^(36,46)^.

#### Healthy start

Dundas et al. used secondary data analysis of existing data sets (Growing up in Scotland (GUS) and Infant Feeding Survey (IFS) to explore breast-feeding, low birth weight, child weight and maternal mental health, with conflicting findings^(46)^. When comparing those who were eligible and receiving HS (R) and those who were eligible but not receiving HS (E), group R were significantly less likely to breastfeed than those in group E in IFS data, but no significant differences were found in GUS data^(46)^. When comparing group R with those nearly eligible (NE) in GUS, maternal mental health was significantly better in group NE^(46)^. When comparing groups R and NE in IFS, group NE had fewer low birthweight infants (group NE = 0·052 % *v*. group R = 0·071 %, *P* = 0·025)^(46)^.

#### EatSF

Wang et al. utilised birth records to assess for associations between the intervention (EatSF) and health outcomes (low birth weight, maternal gestational diabetes, maternal weight gain)^(36)^. They found no significant differences between the intervention and control groups, although it is likely that only a small proportion of the intervention group (∼11 %) received the intervention, due to low programme enrolment in the intervention county^(36)^.

### Qualitative papers

Five qualitative studies explored HS in the UK^(42–46)^, and two explored the FMNP in the USA^(37,48)^ (Table 5).

**Table 5.** Studies considering the impact of fruit and vegetable vouchers: qualitative findings

Paper ID	Scheme, data collection	Main findings
Blumberg 2022^(37)^	Farmers’ Market Nutrition Program (FMNP), open question section in survey. *n* 329	Barriers cited included: Lack of time, interest, knowledge about the programme or access to transport Language barriers, the presence of children, distance to and opening times of the market can make shopping difficult The market is not reliable, sells out or lack variety
Jacobs 2023^(48)^	FMNP, semi-structured interviews with FMNP recipients and staff. *n* 21	Participants (staff and FMNP recipients) supported the underlying concept of FMNP and felt that it has the potential to improve access to F&V FMNP recipients reflected positively on experiences at FM and felt that programme was important One recipient reported using the vouchers to supplement food that they already had when they did not have enough money to buy the foods they needed Staff reported WIC claimants aware of the FMNP asking about it in subsequent seasons Some FMNP recipients expressed a preference for FM produce over supermarket produce due to belief that FM had superior quality/were fresher. Among recipients who did not redeem the coupons, some were unsure where they could be used. Some recipients felt that processes around FMNP could be better standardised between WIC sites Some suggested smaller ‘pop-up’ shops in communities without FM to allow more recipients to use the vouchers.
Dundas 2023^(46)^	Healthy Start (HS), semi-structured interviews with low-income mothers, including those receiving and not receiving paper-based HS. *n* 22 recipients *n* 18 non-recipients	Overall, participants had good understanding about the aims of scheme but confusion around eligibility criteria Some were unaware of the scheme Recipients felt positively about HS Critical about low voucher value and limited eligibility criteria Some saw the vouchers as a health intervention and others as a financial resource Most used vouchers in supermarkets – better value and range of produce, felt more confident that they would be accepted and handled discretely and less likely to check shopping Some used the vouchers to buy more or more expensive F&V No one stated that HS influenced their decision to formula feed or breast-feed, formula feeding mothers felt it was unfair that they did not have any money left over for F&V. Many had tried to breast-feed but were not successful Some saved HS vouchers for emergencies
Lucas 2015^(42)^	HS, in-depth interviews with purposively recruited parents. *n* 107	Many had a smooth application process but some experienced difficulties, and these could be challenging to resolve. The need to re-apply after an infant’s birth was a barrier Issues for those close to the eligibility cut-off HS has impact in three ways: subsidising food already bought, facilitating purchase of greater quantity or variety of F&V and providing a safety net Some felt the vouchers are not worth enough Some reported feeling ‘shame’ as a result of using the vouchers Key vulnerable groups were excluded from the scheme (asylum seekers, non-English speakers and those not accessing healthcare)
McFadden 2014^(43)^	Paper-based HS Focus groups and online consultation with professionals, workshops, focus groups and interviews with low-income parents. *n* 781 (Healthcare Professionals (HCP): *n* 49, Online consultation: *n* 619, Parents: *n* 113)	Eligibility more difficult and discriminatory against low paid working applicants compared with those receiving benefits Some wanted eligibility criteria broadened Limited awareness of the scheme especially for those who do not speak English Challenging application process HS enabled better quality and broader variety of F&V to be purchased Many reported financial benefit rather than change in shopping habits Some continued to buy more F&V after end of scheme Many felt the voucher value needed to increase Greater influence on breast-feeding mothers due to high cost of formula milk Some wanted to be able to use vouchers online Lack of culturally appropriate F&V in supermarkets Some reported stigma associated with the vouchers
Moonan 2022^(44)^	Paper-based HS Interviews with parents and professionals. *n* 42 (Parents: *n* 25, HCP: *n* 11, Commissioners and HS staff: *n* 6)	Some confusion over participating retailers The monetary value of the vouchers was appreciated but felt that this needed to increase
Ohly 2019^(45)^	Paper-based HS Realist interviews with pregnant and postpartum women. *n* 11	HS enabled some women to improve their diet Greater variety of F&V for the whole family (not just intended recipient) HS may reinforce healthy eating Some used the vouches as financial assistance or a nutritional safety net Financial stress may reduce relative importance of healthy eating for some Some used vouchers to stockpile formula during pregnancy No indication that HS vouchers impacted parent’s decision to breast or formula feed

#### Healthy Start

Three studies held interviews with parents^(42,45,46)^, and two undertook qualitative work with both parents and professionals^(43,44)^. Common themes throughout the studies included the way in which vouchers are used: as a financial benefit to subsidise food already being purchased^(42,43,45)^, to increase the quantity or variety of F&V purchased^(42,43,45)^, or as a safety net, to be used to ensure that the family had something to eat^(42,43)^. All five papers found that participants felt the monetary value of the HS vouchers was insufficient^(42–45)^. Issues with the application process and eligibility criteria were highlighted^(42–44,46)^, as well as with awareness of the scheme^(42,43,46)^.

#### Farmers Market Nutrition Program (FMNP)

Jacobs et al. explored participant’s and staff’s experiences of the FMNP using semi structured interviews^(48)^. Blumberg et al.’s, mainly quantitative, study included open survey questions on barriers and enablers of voucher redemption, which were qualitatively analysed^(37)^.

The heterogeneous nature of the interventions explored, the study designs used and the outcomes explored make robust synthesis of the data challenging. Much of the reported data is observational or non-randomised, which limits conclusions that can be drawn and confidence in any quantitative results. This, in part, reflects the challenges of evaluating public health interventions^(63–65)^.

Table 6 presents the GRADE assessment and summary of quantitative findings. Overall, certainty in the evidence was low. There was more consistence of results from qualitative work, with reasonable triangulation of concepts between studies, and, in general, better study quality.

**Table 6.** Summary of findings including GRADE assessment

Outcome of interest	Summary of effect	Number of participants included (number of studies)	Certainty in the evidence (explanation)*
Diet quality	Majority of studies found that F&V vouchers were associated with increased intake of F&V^(33,34,39,41)^, or vegetables alone^(37,38)^. One study found no significant differences between groups^(35)^.	*n* 3122 (7)	Low ⊕ ⊕ 0 0 (serious concerns about methodological limitations and borderline serious concerns about inconsistency)
Fruit and vegetable purchasing (amount spent and/or quantity)	Inconclusive. Two studies report increased spend on F&V associated with the intervention^(40,47)^, and one reported no significant differences^(20)^. One study reported counts of F&V purchased at intervention site but not control sites^(32)^.	*n* 752 individuals, *n* 5165 Households (4)	Low ⊕ ⊕ 0 0 (serious concerns about methodological limitations and inconsistency)
Total food expenditure	Inconclusive. One study reported increased total food expenditure associated with the intervention,^(47)^ and the other found no significant differences^(20)^.	*n* 150 individuals and 4869 Households (2)	Low ⊕ ⊕ 0 0 (serious concerns about methodological limitations and inconsistency)
Food security	Inconclusive. One study found a positive impact of F&V vouchers of food security^(34)^, two reported no significant differences^(35,38)^	*n* 2545 (3)	Low ⊕ ⊕ 0 0 (serious concerns about methodological limitations and inconsistency)
Low birth weight	Inconclusive. One study found significant differences between the intervention and control groups in one dataset but not in another dataset^(46)^. The other found no significant differences between groups^(36)^	*n* 1, 841, 956 (2)	Low ⊕ ⊕ 0 0 (serious concerns about methodological limitations and inconsistency)

Table adapted from Schünemann H, Brożek J, Guyatt G, *et al.* GRADE Handbook: Handbook for grading the quality of evidence and the strength of recommendations using the GRADE approach.: Cochrane Training, 2013. *https://gdt.gradepro.org/app/handbook/handbook.html*, and Murad MH, Mustafa RA, Schünemann HJ, *et al.* Rating the certainty in evidence in the absence of a single estimate of effect. Evid Based Med 2017;22(3):85–87.

‘High: We are very confident that the true effect lies close to that of the estimate of the effect.

Moderate: We are moderately confident in the effect estimate: The true effect is likely to be close to the estimate of the effect, but there is a possibility that it is substantially different.

Low: Our confidence in the effect estimate is limited: The true effect may be substantially different from the estimate of the effect.

Very Low: We have very little confidence in the effect estimate: The true effect is likely to be substantially different from the estimate of effect’ (section 5)^(27)^.

* GRADE Quality of evidence grades, taken from GRADE Handbook^(27)^: Schünemann H, Brożek J, Guyatt G, *et al.* GRADE Handbook: Handbook for grading the quality of evidence and the strength of recommendations using the GRADE approach.: Cochrane Training; 2013. *https://gdt.gradepro.org/app/handbook/handbook.html.*

### Grey literature

Eight grey literature reports have been included in this review (Table 7). It was not possible to formally quality assess these documents due to a lack of detail in the methodological information available. Five reports explored HS^(49,51,52,55,66)^, one explored BSF^(50)^ and two evaluated Rose vouchers^(53,54)^. Amongst the reports focussing on HS, common themes identified included lack of clarity around various aspects of the scheme^(52,55,66)^, issues around access to retailers signed up to HS^(49,51,52)^, the use of HS to increase quantity or variety of F&V purchased^(51,55,66)^ and the need to make changes to the eligibility criteria^(49,51)^. After the introduction of BSF the Scottish government commissioned an evaluation of the scheme. Recipients reported using BSF to purchase a greater quantity or variety of F&V, to reduce financial pressures or as a safety net^(50)^. There were concerns about lack of understanding of some aspects of the scheme, and some felt that eligibility criteria should be broadened^(50)^. In general, the use of a pre-paid card rather than paper vouchers was felt to be a positive change^(50)^.

**Table 7.** Grey literature reports considering the impact of F&V voucher schemes

Document ID and scheme	Study design, location and sample size	Relevant outcomes included	Main findings
Qualitative reports			
Food matters 2012^*^^(51)^, Paper-based Healthy Start (HS)	Qualitative. Participatory workshops (*n* 11) with HS recipients and those who have recently left the scheme, low-income pregnant women and parents of children aged 0–4 years old. England, UK Sample size unknown	Experiences of the scheme Views of those not receiving the scheme	Importance and influence of HS Most stated that HS allowed to experiment more and buy better quality/ larger variety of F&V Some used HS to save money to be spent elsewhere HS had more impact on diets of breast-feeding mothers, due to high cost of formula. Some reported no change in buying habits but relieved financial stress Many parents noticed the difference when child was no longer eligible. Helped to establish healthy habits and provided a ‘nudge’ for parents Awareness of HS Information and promotion of HS and eligibility criteria is inconsistent Eligibility Complicated Particularly challenging for those with changing incomes, those who are self-employed and teenage parents Should be extended to those aged 4–5 years Eligibility being means tested creates delays, including receiving vouchers after a change of address etc Using HS Differences in how retailers let people use the vouchers, i.e. one at a time, checking items, unspent value lost Can be used at self-service checkout- less scrutiny Stigma felt by many Not promoted amongst independent/ local shops - have to go to larger shops, especially challenging in rural areas Difficult to get culturally acceptable F&V Mostly clear what you can buy but some confusion about frozen and tinned foods Mostly vouchers shared amongst the family (some bought for a specific child) HS and infant feeding Behaviour change impact less for formula feeding (FF) families due to high cost of formula Currently, the scheme nudges towards FF and removing formula would nudge towards breast-feeding, although women reported other influences on their decisions. Some started FF sooner than they would have without HS Support for FF makes it easier for some women to remain in education Some pregnant women said they used the vouchers to build up a supply of formula Some thought the availability of formula through the scheme made the decision to FF appear more acceptable
Nottinghamshire 2021^(52)^, Paper-based HS	Qualitative. Families attending a children’s centre. England, UK Sample size unknown	Recipient experiences of the scheme	Lack of clarity around where to use the vouchers Some parents would like to use the vouchers in a wider range of shops Families stated that the vouchers do not cover the cost of formula. Some families chose not to use the vouchers but to give them to others more in need Some families were reluctant to admit that they receive HS due to associated stigma Important to tell recipients to re-apply after changes in circumstances
Mixed methods reports			
Food matters 2017^(53)^, Rose vouchers	Mixed methods before and after. England, UK. 58/121 families receiving vouchers participated in the mid-scheme evaluation and 68/162 families receiving vouchers participated in the final evaluation workshop.	Food consumption (using food diaries) Recipient experiences	Quantitative data Increase in amount of fresh fruit consumed (89 % of adults and 94 % of children) Increase in amount of fresh vegetables (90 % of adults and 95 % of children) Qualitative data Families reported consuming more F&V, with more achieving 5 a day Helped increase variety and try new things Fruits used for snacks Reduced financial stress Some used the money saved to buy other things Some would continue to eat more F&V after the scheme ends but reducing spend elsewhere Some reported positive health outcomes – reduced constipation, increased energy levels, improved skin and weight loss Some felt the vouchers supported behaviour change Markets provide good value Some issues with quality of the F&V In some cases, markets were not convenient, and the cost of transport was prohibitive
Lloyd 2014^(54)^ Rose vouchers	Mixed methods before and after. England, UK. Mothers with a child aged 1–4 received vouchers (*n* 81). Children’s centre and project staff.	Food consumption (using food frequency questionnaire (FFQ) and 24-hour dietary recall) Experiences of mothers and children’s centre staff	Quantitative data Increase in F&V intake (NS in most groups) No change in consumption of ‘unhealthy’ foods No change in proportion of meals that were home cooked, ready meals or eaten out No change in breast-feeding or FF rates Increased spend on F&V and food overall Qualitative data Recipients were happy with range and quality of F&V available at market More culturally acceptable choices Markets were cheaper than supermarket Vouchers used as intended Recipients reported increasing spend on F&V For some, vouchers triggered increased priority being put on F&V Some reported increased intakes of F&V, less ‘junk’ food and more home cooking Some reported more vegetables and more balance meals Some enjoyed being able to experiment more without ‘risk’ Many reported being more aware of healthy eating Children’s centre staff felt that benefits of the vouchers outweigh the additional workload
Liverpool 2022^(66)^ Paper based and digital HS	Mixed Methods. England, UK Discussions with parents and staff from a variety of organisations Focus groups (*n* 2) with health visitors, and staff from voluntary and community sector Interviews with staff from Housing, Public Health, Citizens Advice Bureau and Local Authority Surveys from parents/carers (*n* 14) Sample sizes not reported	Experiences of parents and carers Experiences of healthcare staff (not possible to separate healthcare staff responses from other professionals)	Parents and carers: Positive feedback about scheme and its value to them Understanding that the scheme aimed to improve diets, but varying awareness of details of the scheme including eligibility Many parents did not receive HS until after their baby was born Many found application process straightforward but contacting HS hard All stated that HS was beneficial to them. Some used vouchers as financial assistance and some to improve diet. Generally positive about move to digital scheme and reduced stigma, but some unhappy with the need to check card balance and some found internet access challenging Language could be a barrier to applying Professionals: Highlighted need for consistent messaging around HS Knowledge and understanding of the scheme varied particularly around details and eligibility Digital system has reduced stigma Barriers: digital exclusion, internet access, IT skills, language, competing life pressures, low literacy levels, issues with the website and phone line, cost of phone calls to helpline Digitisation has made it more difficult to support parents with the application Some had concerns around what parents used the vouchers for, but others felt that this was not their concern Access to large, low-cost supermarkets was an issue, with some families lacking transport to get to the shops, and being forced into using local, more expensive shops Since COVID, opportunities to promote HS have reduced
Tavistock 2005^(55)^ Paper-based HS	Mixed methods. England, UK Qualitative feedback at national (*n* 21) and local levels (*n* 112) Quantitative data: Health Care Professionals (HCP) (*n* 32) and recipients (*n* 18)	Recipient and HCP experiences of the scheme	Lack of clarity around eligibility Available written information focused on access to scheme rather than health promotion Most beneficiaries with older children (over one year) used vouchers to buy F&V Over half said they were buying more F&V since the vouchers were introduced HCP reported that target population had poor diets and lack of food preparation skills
CPAG 2015^(49)^ Paper-based HS	Mixed methods, anonymised case studies, qualitative work and policy seminar. Scotland, UK Unclear. Included: Frontline workers Child poverty action group (CPAG) workers Low-income families (*n* 12)	Outcomes from policy seminar	Need to reduce burden of application for recipients and retailers Must have more language options Expensive phone lines and need to re-register after birth of infant are both barriers Some felt BSF should be universal, others that upper age limit should increase to 5 years of age, and others that the focus should be on improving uptake Some felt vulnerable groups should be included automatically or that eligibility criteria should be broadened Some reported stigma around using BSF Difficult to use in rural areas Some felt the list of products included should change – removing formula milk and/ or including other ‘healthy’ foods (i.e. oily fish, grains, etc)
Scottish Government 2022^(50)^ Best Start Foods (BSF)	Mixed methods, depth interviews, survey and secondary data analysis. Scotland, UK Best Start Foods (BSF) recipients (*n* 33) Healthcare professionals (HCP) (*n* 5) Retailers (*n* 9, large and small supermarket chains in urban and rural settings)	Recipient, retailers and HCP experiences of the scheme	Issues with applications for some, went smoothly for many Lack of understanding around some aspects of the scheme Benefits to a prepaid card over vouchers (reduced stigma, does not expire, easier for retailers) Lack of data collection was a missed opportunity Most used the scheme as intended Recipients found drop in value after the child turns one difficult to manage Some felt BSF allowed them to purchase more or a greater variety of F&V Some saved the money spent of F&V to be spent elsewhere Some reported using the card as a safety net, or that it reduced financial pressures and stress Some recipients and HCP felt that BSF increased their awareness of healthy diets and improved budgeting skills Some suggested a need for auto enrolment and increased promotion of the scheme HCP raised concerns about BSF not matching cooking skills or tastes of recipients Some wanted BSF to cover a wider range of food (meat, bread, etc) and non-food (nappies, clothes) items

* The food matters document focusses specifically on the participatory workshops that contributed to one of the peer reviewed papers included in this review, by McFadden *et al.* ^(43)^.

Finally, two evaluations of the Rose voucher scheme were undertaken^(53,54)^. Recipients reported consuming more F&V, some used the vouchers to reduce financial pressures and others to purchase larger quantities or varieties of F&V^(53,54)^. Some recipients felt that the scheme supported healthy habits and that the scheme was likely to change their habits in the longer term, with some reporting improved health outcomes (reduced constipation, feeling healthier, weight loss, improved skin, improved energy levels, improved mental wellbeing), which they saw as being a result of the scheme^(53,54)^.

## Discussion

This systematic review explores the impact of F&V voucher schemes on a range of outcomes. The most commonly included group of outcomes were F&V purchasing and F&V consumption. Overall, F&V voucher schemes did appear to increase fruit and/or vegetable consumption, but confidence in this finding was low. Qualitative data was more consistent. F&V vouchers were used in three main ways; as a financial benefit to subsidise food already being purchased, to increase the quantity or variety of F&v. purchased, or as a safety net, to be used to ensure that the family had something to eat.

There was a lack of consistency of results across the studies included in this review, with different outcomes being considered and some studies finding a positive impact of F&V vouchers whilst others found no significant differences. There are several possible reasons for this. Firstly, evaluating interventions such as F&V voucher programmes is challenging^(64)^. Often, researchers have to utilise existing datasets or use proxies to determine either exposure or outcome variables, which introduces bias into the study. In many of the studies included in this review, estimates were unadjusted for some or all major confounders that could be expected to impact the results^(32,34,35,37–41,47)^, mostly due to the data being unavailable. Studies included in this review used a wide range of methods and data sources, and studied several different populations. Some studies, such as that by Ridberg et al., may have been impacted by the COVID pandemic and the introduction of other assistance schemes that could have diluted the impact of the intervention^(35)^. Others reported large differences in the rates of overestimation and underestimation of food intake between intervention and control groups, which, again, may have impacted their results^(39,41)^. When taken in totality, these factors make it challenging to draw firm conclusions from the available data.

Some studies found that F&V vouchers increased fruit and/or vegetable purchasing^(40,47)^ or consumption^(33,34,37–39,41)^, whilst others found that they made no significant differences^(20,35)^. In the case of the UK-based HS and BSF schemes, vouchers can be used to purchase both cow’s and infant formula milk, as well as F&V, which may have diluted the impact of the vouchers on F&V purchasing and consumption outcomes, particularly in the case of families with infants who are formula fed or not yet fully weaned. This is due to the comparatively high cost of infant formula, which means that it is unlikely that families would be able to purchase both sufficient formula for their child’s needs and F&V with the vouchers provided^(67)^. Overall, when considering the impact of F&V voucher schemes on F&V purchasing and consumption (and associated nutrient intake), the weight of evidence would suggest that, F&V vouchers schemes are likely to increase F&V purchasing and F&V consumption, but to what degree is unclear and confidence in this finding is low. Some studies examining F&V vouchers in older children have found an association between F&V vouchers and increased fruit and/or vegetable purchasing or consumption^(68–72)^.

One concern raised about the HS and BSF programmes, is that, by allowing recipients to use their vouchers to purchase infant formula, the scheme incentivises bottle feeding of infants^(49)^. The only study to explore this outcome found inconclusive results, with a negative association between HS and breast-feeding in one dataset, and no significant difference in another^(46)^. Interestingly, Parnham et al. found that HS recipients spent significantly less on infant formula than eligible non-participants of the scheme^(20)^. Whether the purchase of infant formula should be allowed under HS is a topic that requires careful consideration. The negative health consequences of removing formula (such as the risk of families being forced to ‘water down’ formula under intense financial pressures^(73)^) from HS may be more damaging than the potential positive benefits of more F&V consumption.

The evidence for impact of F&V vouchers on health outcomes was limited and conflicting. Wang et al. found no evidence of association between EatSF and maternal/foetal health outcomes^(36)^, but it is important to note that Wang et al. used proxies to determine intervention status, which meant that only approximately 11 % of the intervention group were likely to be receiving the intervention^(36)^. Dundas et al. explored associations between HS and low birth weight and maternal mental health, and found different results in the different datasets analysed^(46)^.

Impact on food security was also unclear, with two studies reporting no significant differences between intervention and control groups^(35,38)^, and one study finding an increase in food security amongst the intervention group^(34)^. In terms of improving food security, an alternative to F&V vouchers could be a cash benefit. There is some debate about the benefits of vouchers compared with cash benefits and the impacts of these on their intended outcome, with much of the evidence coming from developing countries^(74)^. Whether cash or voucher benefits have more impact is likely to be context and intervention specific^(74)^, and it is therefore difficult to predict whether cash benefits may offer any additional positive impact over vouchers.

In contrast, the qualitative data included in this review are more cohesive, with striking similarities found across several different studies, and triangulation of these views between recipients and HCP in some cases. Most qualitative studies included in this review explored HS. Three main ways in which HS vouchers are used are highlighted in both the peer reviewed and the grey literature: subsidising food that would have been bought already^(42,43,45)^, buying greater quantity or variety of F&*v*.^(42,43,45)^ and acting as a safety net to prevent families from going hungry at times of crisis^(42,45)^. It was clear that there were issues with the paper-based HS scheme, with difficulties around applications and eligibility frequently mentioned^(42–44)^, as well as an acknowledgement that the voucher value has been insufficient to keep pace with rising food costs^(42–45)^. HS has recently transitioned into a digital scheme with a prepaid card, which can be used at any retailer which accepts MasterCard^(56)^. There has also been a small uplift in the voucher value, to £4·25 per week for pregnant women and children aged 1–4 years, and £8·50 per week for infants aged 0–1 year^(56)^. Whilst this is likely to have resolved some of the issues highlighted in the literature, the transition has been far from smooth for many^(46,75)^, and the increase in HS value has not kept pace with rising food costs^(76)^. Additionally, the eligibility criteria have not significantly changed, so many issues around the exclusion of vulnerable groups are likely to remain, and gaps in eligibility (for example for children aged 4–5, before they may become eligible for free school meals upon starting school^(77)^). A review published in 2016 exploring the use of vouchers in the HS and WIC programmes, found that vouchers were used to improve dietary quality, and to reduce food expenditure^(78)^, both themes also found in this review.

Some alternatives to F&V vouchers have been explored in the literature, for example, F&V or produce boxes^(79)^. Fischer et al. explored the impact of a fortnightly F&V box, delivered to families with preschool children, alongside nutrition education^(79)^. Whilst satisfaction with the programme was high, impact on F&V intake and food insecurity was uncertain with most changes failing to meet statistical significance^(79)^. A recent review exploring produce prescription interventions found that F&V boxes were acceptable to recipients, and some evidence for increased F&V consumption, but concede that evidence in this area could be improved^(80)^. Whilst F&V boxes may be appealing in some respects, removing concerns about how vouchers are used and perhaps encouraging families to try new foods, they are logistically challenging to organise, and may be wasted if families receive produce that they do not like or do not know how to use. One issue highlighted in this review is, for some, a lack of understanding of nutrition and food preparation knowledge hinders attempts to improve diets for some families. One French study offered nutrition education workshops alongside F&V vouchers. They found that changes in F&V consumption were not associated with attendance at a workshop^(72)^. In their recent scoping review, Greatorex Brooks et al. concluded that educational elements to F&V prescription programmes needed further exploration, in order to better understand their contribution (or not) to the programme’s success^(81)^.

All of the studies included in this review examined targeted interventions designed to support those on low incomes, with means tested eligibility criteria. Interestingly, the level of financial hardship needed to qualify differs across the schemes. HS, Rose vouchers and Sainsbury’s top up vouchers have stringent eligibility criteria, whilst BSF has slightly more generous criteria. WIC eligibility (and therefore FMNP and EatSF eligibility) varies by state up to a maximum of 185 % of the federal poverty guidelines^(82)^, resulting in 48 % of children under 5, pregnant and postpartum women being eligible for WIC in 2021^(83)^. Universal provision was raised by some participants as a potential improvement to F&V vouchers^(49)^ and was recommended by the UK Faculty of Public Health in January 2024. There are some benefits to this approach, reduced administrative load assessing eligibility, perhaps a reduction in stigma associated with the vouchers and an emphasis on the importance of healthy diets. However, increased costs of the schemes may be off putting to policy makers. This debate raises the question of whether population or targeted approaches are more successful in terms of improving population health. Clearly, the answer to the question is likely to differ depending on the population, the intervention and the desired outcome^(84)^. There is some evidence that population level health interventions have the potential for positive impact on outcomes^(85–88)^. Whether this would be the case in this context is unclear currently, it may be that a combination of population level and more targeted approaches offers the most effective approach^(86)^. These may include focusing on specific geographic areas of higher deprivation to increase uptake or testing extended eligibility criteria in such areas. Further targeted interventions could include cooking sessions during school holidays or mobile vans that provide fresh produce to areas where fewer affordable options are available.

### Strengths

This review took a systematic approach and used broad inclusion criteria resulting in the exploration of a range of outcomes. Another strength is the inclusion of quantitative, qualitative and mixed methods studies, which has allowed exploration of the impact of F&V vouchers in a more holistic way. Finally, the inclusion of grey literature has ensured that important findings were not excluded by virtue of not being published in an academic journal, limiting publication bias.

### Limitations

Interventions that met inclusion criteria were only found in two geographic regions, the USA and the UK, which limits generalisability of these findings to other parts of the world. Most studies exploring HS looked at the paper-based scheme, and so do not necessarily reflect the current, digital scheme. The wide variety of study designs, methods and outcome measures make it difficult to draw direct comparisons between some of the findings, particularly the quantitative outcomes, and necessitated a narrative approach to synthesis. No quantitative evidence was found for some outcomes included in the review inclusion criteria as follows; nutritional biomarkers, childhood or maternal weight status or childhood healthcare contacts or healthcare utilisation, although many of these topics were explored in the qualitative data. Finally, the review is limited by the quality of evidence available in the literature. Whilst not a limitation of the methods of this review, the majority of the studies included were of designs that are lower in the hierarchy of evidence, and many were not able to control for confounding or had to use proxies to determine intervention or outcome status. This is not unexpected given the type of intervention and need to be pragmatic and make use of available data. However, it does limit the confidence in the findings of the studies, and, in turn, this review.

### Conclusions

In conclusion, it is possible that F&V vouchers increase F&V intake, although certainty of evidence is low. It is likely that F&V vouchers have some positive benefits, and they seem to be perceived in a positive light by recipients and staff. It is possible that F&V vouchers may have more significant impacts on certain groups—for example for families with breast rather than formula fed children in the HS/BSF schemes, due to the high cost of infant formula. The food purchasing behaviours of the recipient are also likely to have an impact on the impacts of the scheme, with those using vouchers to subsidise existing choices likely to have different experiences to those choosing to buy more, or more varied F&V. There is a potential for positive mental health impact through reduced financial stress regardless of the approach used when redeeming the vouchers.

This review highlights some of the difficulties that researchers face in evaluating the impact of public health measures to improve population health. More, high-quality research is required to better understand the impacts of F&V vouchers on outcomes. This includes research which considers uptake of the schemes, captures outcomes consistently with longer follow-up, enables researchers to control for confounding and understanding the experiences of people using digital schemes.

It is clear that there are significant operational challenges associated with voucher schemes. Several factors are important to consider when designing F&V voucher schemes; eligibility criteria, accessibility of scheme, voucher value and stigma associated with the scheme, amongst others. It is important that the voucher value of F&V schemes keep pace with food costs and are taken up by those eligible for it. Evaluation of the scheme could help identify potential changes required to ensure that the target population of pregnant women and families with young children benefit from the voucher scheme.
